# Modern perspectives in Proof Theory

**DOI:** 10.1098/rsta.2022.0020

**Published:** 2023-05-29

**Authors:** J. P. Aguilera, F. Pakhomov, A. Weiermann

**Affiliations:** ^1^ Institute of Discrete Mathematics and Geometry, Vienna University of Technology, Wiedner Hauptstraße 8–10, 1040 Vienna, Austria; ^2^ Department of Mathematics, University of Ghent, Krijgslaan 281-S8, B9000 Ghent, Belgium; ^3^ Division of Mathematical Logic, Steklov Mathematical Institute of the Russian Academy of Sciences, Ulitsa Gubkina 8, 117966 Moscow, Russia

## 1. Introduction

The articles collected in this volume form a snapshot of the state of Proof Theory at the beginning of the year 2023.

Proof Theory is a branch of mathematical logic originated by Hilbert in the early 1900s. His goal was to devise a theory of mathematical proofs, itself making use of tools from Mathematics. Such a theory would identify abstract properties of proofs and trace the limits of provability. A particular question of interest was ‘is there a proof of a contradiction from a given set of axioms T?’ or otherwise stated: ‘is T inconsistent?’ Hilbert’s goal with Proof Theory was to show, via combinatorial methods, that no mathematical proof could have a contradiction as its conclusion.

Gödel’s [1] incompleteness theorems showed that Hilbert’s goal could not be attained in a strict sense: for sufficiently strong, consistent theories T, any mathematical proof of the fact that T can only be carried out in a mathematical theory $T^{\prime }$ stronger than T. In particular, Gödel’s theorems applied to theories such as first-order and second-order arithmetic, i.e. PA and $Z_{2}$.

Ever since, the Proof Theory has focused, in one way or another, on attempting to avoid the limitations of Gödel’s incompleteness theorems. These attempts have led to rich theories of proofs, metamathematics and unprovability results of various kinds. We mention two of them.

### (a) Reverse Mathematics

Reverse Mathematics addresses the question ‘which mathematical axioms are necessary in order to prove a given theorem φ?’ Thus, while we might not be able to give a completely satisfactory proof that the axioms needed to prove φ are infallible, we might reach a better understanding of them, their relationship with other axioms and the logical content of φ. This may lead to generalizations, optimal proofs and independence results. The programme was explicitly initiated by Friedman [2] and has had high momentum ever since.

Technically, Reverse Mathematics is carried out as follows: one begins by fixing a base theory T over which to carry out the analysis. This theory must be weak enough so that the target theorem φ is not provable in T; often one considers $T=RCA_{0}$ (Recursive Comprehension), the theory whose characteristic axioms are the induction schema restricted to $\Sigma_{1}^{0}$ formulas with second-order parameters, as well as the axiom asserting that all recursive sets exist. One then must (i) derive the theorem φ from some stronger set of axioms A and (ii) derive the axioms A from the theorem φ, establishing the logical equivalence of A and φ, i.e. the sufficiency and necessity of the axioms for a proof of φ.

An early observation was that most theorems of ‘everyday Mathematics’ line up in strength with one of five subsystems of $Z_{2}$, called the ‘Big Five’. These are $RCA_{0}$, ${WKL}_{0}$ (the result of extending $RCA_{0}$ by weak König’s Lemma, the assertion that every infinite binary tree has a branch), $ACA_{0}$ (Arithmetical Comprehension, the second-order counterpart of PA asserting that all arithmetical sets relative to a second-order parameter exist), $ATR_{0}$ (Arithmetical Transfinite Recursion, asserting that every arithmetical operator can be transfinitely iterated along any well-order) and $\Pi_{1}^{1}-CA_{0}$ ($\Pi_{1}^{1}$-Comprehension, asserting that every $\Pi_{1}^{1}$ set exists). Aside from work in actual Reverse Mathematics, research within the field often concerns the abstract theory of Reverse Mathematics itself, the study of the axiomatic systems for its own sake, the study of models of the theory and the relation between subsystems of $Z_{2}$ and other systems of first- or higher-order arithmetic or of set theory. A general reference for the subject is Simpson [3]. We also refer the reader to Dean and Walsh [4] for an overview of the historical context of the development of Reverse Mathematics going back to Poincaré.

### (b) Ordinal Analysis

Gentzen [5] gave the first proof (two, in fact) of the consistency of number theory (more precisely, PA). By Gödel’s theorem, such a proof must necessarily go beyond the means of arithmetic itself, and the way this happens in Gentzen’s proof is interesting. The proof follows a direct, explicit, inductive construction which reduces a putative proof π of a contradiction to a simpler such proof, eventually producing a proof so simple that it can be verified directly π could not exist. Each step of the induction is simple enough that it can be verified in very weak theories, such as Primitive Recursive Arithmetic. However, the ordering over which the induction has been carried out is very long, namely, of order-type

$$\varepsilon_{0}=\sup\{\omega ,\omega^{\omega },\omega^{\omega^{\omega }},\ldots \},$$

where ω denotes the order-type of the natural numbers. The explanation behind the possibility of Gentzen’s proof is that PA does not prove that the ordering $\varepsilon_{0}$ is well-founded, and thus, that induction along it is possible.

A longstanding goal of Proof Theory has since been to prove analogues of Gentzen’s theorem for stronger and stronger theories. This gives rise to the concept of the $\Pi_{1}^{1}$-norm of a theory,

$$|T{|}_{\Pi_{1}^{1}}=\sup\{\alpha :T⊢\mathrm{wo}(A)\text{ for some }A\text{ isomorphic to }\alpha \}.$$

By Spector’s $\Sigma_{1}^{1}$-boundedness theorem, $|T{|}_{\Pi_{1}^{1}}<\omega_{1}^{ck}$ whenever T is $\Sigma_{1}^{1}$-sound.

In the decades following Gentzen’s work, several such analogues have indeed been proved, most notably by Takeuti [6], who obtained a consistency proof for the system $\Pi_{1}^{1}-CA_{0}$, and Rathjen [7], who obtained a consistency proof for the system $\Pi_{2}^{1}-CA_{0}$. Arai [8] has recently announced an ordinal analysis of KP augmented with the schema of $\Pi_{1}$-collection. We refer the reader to Rathjen [9] for a more detailed overview.

The recent work in the field aims at extending the scope of Ordinal Analysis in various ways. Arai [10] presents a form of un-effectivized ordinal analysis which studies the countable ordinals whose existence is provable in ZFC. Aguilera and Pakhomov [11] study extensions of the notion of $|T{|}_{\Pi_{1}^{1}}$ to higher complexity classes.

## 2. Tropes in this volume

Various ideals of present-day Proof Theory occur repeatedly in the articles presented in this issue, among which we have identified the following:

(i) Higher-type analogues of well-orders, such as dilators and ptykes, and their use for (i) abstract analyses of theories taking into account their consequences of complexity greater than $\Pi_{1}^{1}$, and (ii) strengthening combinatorial independence results into reverse-mathematical equivalences.
(ii) Reverse Mathematics beyond the ‘Big Five’ subsystems, targeting instead theories stronger than the Big Five, weaker than them (possibly constructive theories), or in between them.
(iii) Connections between constructibility, intuitionism and ‘concrete’ proofs on the one hand and classical systems (or classical Mathematics) on the other hand.

These three themes challenge classical attitudes held by proof theorists in the past, and we expect them to gather even more momentum in the future. The content of this issue should serve as a good starting point for future work, particularly for future young researchers in the field. Because of this, the articles have mostly been written with an open-ended attitude and indicate various open problems.

Before moving on, let us mention some promising Modern Perspectives in Proof Theory, which in an ideal world would have also been covered in this issue.

### (a) Provability logic and its models

Provability logic studies the abstract properties of the provability relation. A good source for the subject is Boolos [12]. By using the tools of modal logic, one can state simple facts about it, such as Gödel’s incompleteness theorem:

◊⊤→◊◻⊥,

an instance of the more general Löb axiom, ◻(◻ϕ→ϕ)→◻ϕ. Solovay [13] proved that the provability logic of Peano Arithmetic can be axiomatized by Kripke’s axiom K, Löb’s axiom and the rules modus ponens and necessitation. The resulting modal system is called GL. Segerberg [14] proved the relational completeness of GL. Solovay’s proof is general and works for other systems such as ZFC, but it notably fails for systems which are much weaker than PA and/or constructive. A solution to the problem of characterizing the provability logic of Heyting Arithmetic has recently been announced by Mojtahedi.

**Question 2.1.**

What is the provability logic of $S_{2}^{1}$?

A related family of problems concerns the topological interpretations of provability logic and of graded provability logics. In particular, the following well-known question is open:

**Question 2.2.**

Suppose that V=L and that for every n∈N, there is a $\Pi_{n}^{1}$ indescribable cardinal $\kappa_{n}$. Is GL complete for the canonical GLP-spaces $\left([1,\kappa_{n}],\tau_{n+1}\right)$?

We refer the reader to Bagaria–Magidor–Sakai [15] or to Bagaria [16] for background on the question, and in particular for the definition of $\tau_{n+1}$, and to Beklemishev and Gabelaia [17] for more background on the subject.

### (b) Reverse Mathematics beyond $Z_{2}$

Recent work has focused on attempts at carrying out reverse-mathematical analyses of statements beyond the reach of $Z_{2}$. Such work not only escapes the paradigm of the Big Five subsystems but also the language of Second-Order Arithmetic altogether, perhaps reaching into the wild zoo of subsystems of Third-Order Arithmetic, or even beyond.

Not many natural examples are known of theorems which can be easily stated in the language of Second-Order Arithmetic but not proved in $Z_{2}$. One such example is Borel Determinacy which—by the work of Martin [18]—is provable in ZFC, but—by the work of Friedman [19]—not in ZC. Friedman’s work also shows that $\Sigma_{4}^{0}$-determinacy is not provable in $Z_{2}$. Reverse-mathematical characterizations of $\Sigma_{4}^{0}$-determinacy have been carried out by Hachtman [20] and Aguilera [21]. The work of Schweber [22] and Hachtman [23] studies natural determinacy assertions which can be stated in the language of Third-Order Arithmetic and identify the subsystems necessary for the proof, illustrating some of the differences which arise in Higher Reverse Mathematics. Aguilera [24] and Aguilera and Welch [25] similarly study determinacy principles which can be stated in the language of Third-Order Arithmetic, but whose proofs require the use of large cardinals.

**Question 2.3.**

Are there other examples of theorems from the mathematical literature which can be stated in the language of Second-Order Arithmetic but not proved in $Z_{2}$?

Notable and promising work on Reverse Mathematics beyond the language of Second-Order Arithmetic has also been carried out by Normann and Sanders. We mention the articles [26–28].

### (c) Structural Proof Theory

Even in 2023, a great deal of research on Structural Proof Theory is being carried out, some of it following foundational inclinations similar to those of this issue. A topic of high momentum currently is infinitary and cyclic proofs.

We mention an old problem in this area, which has been open for more than 50 years and has come to be known as Kreisel’s conjecture.

**Question 2.4. (Kreisel)**

Let PA be Peano Arithmetic axiomatized with the successor induction schema and identity axioms. Suppose that ϕ(x) is a formula such that, for some k∈N, $\phi (\bar{n})$ has a Gentzen-style proof of length ≤k for each n∈N. Must it be the case that PA⊢∀x ϕ(x)?

Many partial results have been achieved towards this conjecture. We mention Baaz and Wojtylak [30] and Hrubeš [31], the latter of which has shown the conjecture to be true if PA is axiomatized with the least-number principle instead of successor induction.

## 3. Individual papers

Let us now give a brief overview of the issue, together with some of our thoughts and comments on future directions of research and open questions. The reader might notice the recurrence of the three themes mentioned in §2 and how they interact with one another.

### (a) Weak set theories in foundational debates [32]

In what is without a doubt the best possible starting point for our issue, Mathias’s article presents a wonderful discussion on the history of attitudes, thoughts and approaches to logic and foundations which slowly morphs into an exposition of provident sets and gentle functions, their properties and the system Provi. This is a weak subsystem of set theory embodying the existence of ω-jumps. Indeed, in the unpublished work, Dorais has shown that ${ACA_{0}}^{+}$ is bi-interpretable with Provi + V=HC. Moreover, if one adds the principle of Mostowski collapse, then one obtains a system bi-interpretable with $ATR_{0}$.

Mathias’s article surveys work from Bowler and Mathias [33], Mathias [34] and others, on this subject, and presents it in a way which encourages the reader to tackle some of the many remaining open questions and research projects. A topic of particular importance is that of forcing over provident sets and extensions of Provi, as well as the notion of a *privileged theory*. A theory is *privileged* if it is preserved by set forcing of models M via (generic) filters G which meet every dense set in M. Clearly, ZFC is privileged, as is Provi, by Mathias [34]. Friedman’s [35] Power Kripke-Platek theory is shown to be privileged here. On the other hand, Zermelo set theory is known not to be privileged (see Mathias [36]), though attempts at fixing this issue give rise to the notion of a *lune*, which is also discussed in this article. A question which appears to remain open and which was stated in Mathias [34] is whether it is possible to force over a model of KP and have a set-generic extension satisfy more separation than the ground model.

Mathias’s article ends with a discussion on stratification and New Foundations, and with an anecdote.

### (b) Metric fixed point theory and partial impredicativity [37]

In this article, Fernández-Duque, Shafer, Towsner and Yokoyama study the Reverse Mathematics of the well-known fixpoint theorems of Caristi [38] and of Preiß-Crampe and Ribenboim [39]. These are interesting theorems to study in Reverse Mathematics because they refer to arbitrary functions on complete (ultra-)metric spaces and thus cannot be immediately formalized in the language of Second-Order Arithmetic. The (reasonable) solution taken by the authors is to restrict to classes of formulas and spaces which can be coded by countable objects in a reasonable way. In particular, the authors study the Caristi fixpoint theorem for Borel or Baire functions and prove an equivalence with Towsner’s leftmost path principle, which lies strictly between $ATR_{0}$ and $\Pi_{1}^{1}-CA_{0}$ in strength.

According to the folklore parlance of mathematicians, Caristi’s fixpoint theorem is equivalent to Ekeland’s variational principle, the latter of which has been analysed in a similar way by three of the authors in [40] and shown to be equivalent to $\Pi_{1}^{1}-CA_{0}$. It is thus interesting that the natural formalization of Caristi’s fixpoint theorem in [37] is strictly weaker. Perhaps an analysis of the full Caristi theorem over a suitable subsystem of Third-Order Arithmetic along the lines of the work mentioned in §2b would shed light into its logical relationship with Ekeland’s principle. The results of [40] and [37] do show, however, that the two theorems are logically equivalent when restricted to compact spaces and continuous potential. The authors also raise the question of the reverse-mathematical status of Caristi’s fixpoint theorem restricted to Baire-1 functions, and in particular:

**Question 3.1. [37]**

Is the Caristi fixpoint theorem for Baire-1 functions provable in $ATR_{0}$?

### (c) Proof Theory and Non-smooth Analysis [41]

Proof mining as pioneered over the years by Kohlenbach and his collaborators concerns proof-theoretic applications to mainstream mathematics. It aims at extracting concrete information (like effective bounds or rates of convergence) from non-constructive proofs in analysis.

This programme has been very successful so far, and a plethora of results has been obtained. Kohlenbach and collaborators on the one hand devised general logical meta-theorems which allow for bound extraction in general contexts, and on the other hand, they successfully applied proof mining to given proofs from the literature.

The current article addresses both issues. In a first step, it covers general results about the proof-theoretic content of monotone and m-accredetive set valued operators. In a second step, this material is used to study the Brezis–Haraux theorem. Two quantitative versions of this theorem are obtained, depending on whether the proof uses the so-called uniform regularity (leading to simpler bounds) or so-called weakly uniform regularity (still allowing for effective bounds) in a critical way.

### (d) On the decomposition of WKL!! [42]

This article properly belongs to the field of *Constructive Reverse Mathematics*. As in usual Reverse Mathematics, the goal of the program is to identify the collection of axioms necessary to prove a given theorem, the difference being that in this case the base theory has been replaced by a constructive system, potentially allowing for a finer analysis.

Fujiwara and Nemoto study a form of the well-known weak König lemma (WKL) over an ‘effectivization’ $EL_{0}$ of Toelstra’s Elementary Analysis whose induction schema has been restricted to $\Sigma_{1}^{0}$ formulas. In this context, Ishihara [43] has previously shown that the weak König’s lemma can be decomposed (over $EL_{0}$) as a logical principle (namely, a fragment of De Morgan’s law $\Sigma_{1}^{0}$-DML) and a choice principle denoted $\Pi_{1}^{0}-{AC}^{\vee }$.

The authors study a version of weak König’s lemma with a uniqueness hypothesis introduced by Moschovakis [44] and ask whether a similar decomposition is possible. They prove that the system WLK!! can be decomposed (over $EL_{0}$) as the union of *two* logical principles (namely, $\Pi_{1}^{0}$-DML and a version of the double-negation shift denoted $(\Pi_{1}^{0}\vee \Pi_{1}^{0})-$DNS${\;}^{0}$), plus *two* choice principles (namely, $\Pi_{1}^{0}-{AC}^{\vee }$ together with an additional choice principle denoted dn-$\Pi_{1}^{0}-{AC}^{\vee }$). While they mention various underivability results between the principles, it is left open whether the reduction is stated in a redundant way, i.e.:

**Question 3.2. [42]**

Does $\Pi_{1}^{0}$-DML imply $\left(\Pi_{1}^{0}\vee \Pi_{1}^{0}\right)$-DNS${\;}^{0}$ over $EL_{0}$?

**Question 3.3. [42]**

Does dn-$\Pi_{1}^{0}-{AC}^{\vee }$ imply $\Pi_{1}^{0}-{AC}^{\vee }$ over $EL_{0}$?

A natural direction of future research is to identify similar decompositions of stronger classical axioms in terms of logical principles plus set-existence, induction or choice axioms, over constructive systems.

### (e) Classical consequences of constructive systems [45]

Intuitionistic logic is a subsystem of classical logic. However, reasonable attempts at doing actual mathematics over intuitionistic logic require selecting an axiomatic system from which one can begin reasoning. Some of these—and, notably, Brouwer’s intuitionistic analysis—are actually incompatible with classical logic. It is natural then to attempt to reconcile constructive mathematics with classical logic as much as possible and, in particular, to ask which consequences of constructive systems are admissible from a classical point of view. While most readers will have encountered examples of theorems from classical mathematics which have to be modified to become intuitionistically provable, perhaps not that many have been in the opposite situation.

In this article, Moschovakis presents a collection of earlier results, as well as some new ones. Topics covered include theorems of analysis in constructive systems which (perhaps after the addition of a new hypothesis) are also provable classically; choice principles which become provable over a system of intuitionistic mathematics; and the minimum classical extensions of intuitionistic systems (perhaps relative to a given classical model of the system).

Theorem 5.2, which asserts the validity in intuitionistic predicate logic of various identities related to unique quantification, has very interestingly found an application as a step in the proof in Moschovakis and Moschovakis [46] of the natural extension of the Strong Spector–Gandy theorem to the higher analytical pointclasses $\Pi_{2n+1}^{1}$ under Projective Determinacy.

### (f) Logics and admissible rules of constructive set theories [47]

By a classical result of de Jongh [48], the propositional principles valid in Heyting arithmetic are precisely the tautlogies of intuitionistic propositional logic. Later Leivant proved that the quantifier logic of Heyting arithmetic coincides with first-order intuitionistic logic [49]. A different generalization of de Jongh’s Theorem is a result of Visser [50] that the admissible propositional rules of Heyting arithmetic are precisely the same as the admissible rules of intuitionistic propositional calculus. In this article, Iemhoff and Passman prove some new results about the logics and admissible rules of constructive set theories as well as providing a survey of the previous results in this area.

The central recent achievements are that both quantified logic (as shown by Passman [51]) as well as the admissible propositional rules (a result presented in this article) of many standard constructive set theories simply coincide with quantified intuitionistic logic and admissible rules of intuitionistic propositional calculus, respectively. The set theories covered by these results are IKP, BCST, ECST, ${CZF}_{ER}$, CZF and CZF+AC. An important technical construction presented in this article that allowed the classification of the admissible propositional rules for many of the mentioned theories in fact showed a general phenomenon: almost any intuitionistic set theory built from certain standard axioms will have the same admissible propositional rules as intuitionistic propositional logic.

This article lists a large number of open questions. One of the open problems in the area is to find first-order logic and admissible propositional rules for IZF. Note that the fact that first-order logic of IZF is larger than just intuitionistic first-order logic is a classical result of Friedman and Ščedrov [52].

### (g) Ordinal Analysis and the set-existence property for intuitionistic set theories [53]

The standard fact about Heyting arithmetic HA is that it has the number existence property: whenever HA⊢∃xφ(x), for some n, $HA⊢\varphi (\underline{n})$, where $\underline{n}$ is the numeral of n, i.e. the term

$$\underset{n\text{ times}}{\underbrace{1+\ldots +1}}.$$

In the case of set theories, of course, there is no analogue of numerals for sets. A theory T is said to have the existence property if whenever T⊢∃xφ(x), then there exists formula ψ(x) such that T⊢∃!x(ψ(x)∧φ(x)). In this article, Rathjen proves that two natural weakenings ${CZF}_{P}$ and ${CZF}_{E}$ of CZF have the existence property. It was previously known that the existence property fails for CZF.

The key feature that makes it hard to prove existence property for these theories is the schemata of collection. By a classical result of Myhill, even ${IZF}_{R}$ has the existence property, note that ${IZF}_{R}$ is a variant of intuitonistic Zermelo–Fraenkel set theory based on replacement instead of collection.

Rathjen used methods of ordinal analysis to establish the existence property for the mentioned theories. Namely, due to a previous result of the study by Rathjen [54], the existence property for ${CZF}_{P}$ and ${CZF}_{E}$ is implied by $\Sigma^{P}$ and $\Sigma^{E}$ existence properties for the theories ${IKP}_{P}$ and ${IKP}_{E}$, respectively. Rathjen then applied the methods of infinitary proof theory for the Kripke-Platek set theory to give an ordinal based characterization of provably total $\Sigma^{P}$ and $\Sigma^{E}$ functions of ${IKP}_{P}$ and ${IKP}_{E}$, respectively, which allows him to obtain the desired existence properties.

### (h) Some independence results related to trees [55]

A highlight of proof theory is concrete independence results for formal systems for reasoning about mathematics. Gödel’s original incompleteness theorem concerned a rather metamathematical statement. Gentzen’s result on the unprovability of the transfinite induction up to $\varepsilon_{0}$ could be regarded as a first example of concrete incompleteness. Later, Paris and Harrington [56] and Kirby and Paris [57] supplied even more concrete independence results via Ramsey theory and combinatorial principles that are equivalent to the primitive recursive well-orderedness of $\varepsilon_{0}$.

Friedman pioneered independence results are related to well-quasi orderings, and this article provides a collection of complementary results about finite trees.

In §§2–4, finite trees are related to monotonicity properties of arithmetical terms and terms for functions from the extended Grzegorczyk hierarchy. A new facet in the discussion is the use of numerical values as complexity measures instead of the more technical notion of number of symbols in a given term. The authors provide proofs for folklore results in the new formulations, and they also give proofs for new variants of well-quasi-orderedness principles which have not been considered before.

In §5, results for one to one tree-order-preserving embeddings of finite trees are discussed. It turns out that in this context, finite trees are basically equivalent to binary trees. Moreover, for binary trees, embeddings and inf-preserving embeddings are the same thing.

Resulting independence results therefore have the strength of the primitive recursive well-orderedness of $\varepsilon_{0}$. In the last section, they discuss finite labelled trees with respect to the Montalbán embedding.

The resulting Kruskal theorem is shown to be equivalent to $ATR_{0}$ using a well-ordering principle which goes back to Friedman.

### (i) The uniform Kruskal theorem: between finite combinatorics and strong set existence [58]

In their recent article, Freund, Rathjen and Weiermann [59] formulated a uniform Kruskal theorem that is a natural generalization of Kruskal tree theorem. One of the variants of Kruskal theorem could be equivalently stated as the principle that certain natural term orders generated by a well-quasi-order set of functional symbols are well-quasi-orders. Uniform Kruskal theorem from [59] extends this to a broader class of term orders, where the functional symbols instead are denotations from some well-quasi-ordering principle F, i.e. an operator mapping each well-partial-order P to a well-partial-order consisting of denotations with parameters from P. Equivalently this term order for a well-quasi-ordering principle F could be defined as the ω-th iteration $F^{\omega }(\emptyset )$ of F. The result of [59] was that the generalized Kruskal theorem is equivalent to $\Pi_{1}^{1}{\text{-CA}}_{0}$.

In this article, Freund and Uftring investigate what base theory is required to prove this equivalence. Namely, they proved that ${RCA}_{0}+ADS$ is sufficient to prove the equivalence between $\Pi_{1}^{1}{\text{-CA}}_{0}$ and uniform Kruskal theorem (ADS is the principle asserting that any infinite linear order either contains an infinite ascending chain or an infinite descending chain). And at the same time, they establish that over ${RCA}_{0}$ the uniform Kruskal theorem does not imply $\Pi_{1}^{1}{\text{-CA}}_{0}$.

The proof of the fact that uniform Kruskal theorem implies $\Pi_{1}^{1}{\text{-CA}}_{0}$ is quite involved and indirect: it relies on a result of Freund that $\Pi_{1}^{1}\text{-}{\text{CA}}_{0}$ is implied by Bachmann–Howard well-ordering principle, which in turn had been obtained by a rather advanced proof-theoretic techniques.

**Question 3.4.**

Is there a simple direct proof of $\Pi_{1}^{1}{\text{-CA}}_{0}$ in ${RCA}_{0}+ADS+$ uniform Kruskal theorem?

Also it would be interesting to find some other natural well-quasi-ordering principles equivalent to stronger theories, such as $\Pi_{2}^{1}{\text{-CA}}_{0}$.

### (j) The spectrum of $\Pi_{3}^{1}$-soundness [60]

This article studies an invariant-like object associated to a theory T which the authors denote $O_{3}^{1}(T)$. Rather than focusing on particular ‘natural’ theories, as is commonplace in the field, the work takes a more abstract approach and attempts to derive general results about T and in particular quantify its degree of soundness. Aguilera and Pakhomov’s goal is to extend the classification theorems of their earlier work [11] to the realm of $\Pi_{3}^{1}$. There, the authors defined the ordinal $o_{2}^{1}(T)$ as the least ordinal α such that D(α) is illfounded for some pre-dilator D such that T⊢dil(D).

Accordingly, $O_{3}^{1}(T)$ is defined as the collection of all dilators D such that P(D) is well-founded for every T-ptyx P, i.e. every recursive (2-)pre-ptyx P such that $T⊢{\mathrm{ptyx}}_{2}(P)$. Thus, $O_{3}^{1}(T)$ serves as a measure of how close T is to being $\Pi_{3}^{1}$-sound: the larger $O_{3}^{1}(T)$ is, the harder one needs to search to find a counterexample to the $\Pi_{3}^{1}$-soundness of T.

The authors begin by recalling various concepts and results from the theory of ptykes. While these facts were already explicitly or implicitly covered in the work of Girard [61] and Girard-Ressayre [62], proofs are included for convenience. Seven classification theorems are proved, all recasting various soundness properties of T in terms of whether certain dilators D belong to $O_{3}^{1}(T)$. As mentioned in the article, these results all extend to the other classes $\Pi_{n}^{1}$ without much trouble.

The last section of the article has a more exploratory nature and raises the question of characterizing the sets of dilators of the form $O_{3}^{1}(T)$ for some recursively enumerable extension T of $ACA_{0}$. Partial progress is made by characterizing the sets of the form $O_{3}^{1}(T)\cap \Delta_{1}^{0}$ for some recursively enumerable extension T of $ACA_{0}$, but the problem is open otherwise. Unlike the results of the earlier sections, this argument relies on the work of the authors [63] on $\Pi_{2}^{1}$ proof-theoretic analysis, and thus, the corresponding questions for $O_{n+4}^{1}(T)$ and $O_{n+4}^{1}(T)\cap \Delta_{1}^{0}$ are open. The *$\Pi_{2}^{1}$-Spectrum Conjecture* from [11] asserts that the following question has a positive answer:

**Question 3.5.**

Let α be an ordinal. Are the following equivalent?

(i) $\alpha =o_{2}^{1}(T)$ for some recursively enumerable extension T of $ACA_{0}$, and
(ii) $\alpha =\omega^{\alpha }$ and α is $\Sigma_{1}^{1}$-definable.

Even phrasing the corresponding question for $O_{3}^{1}(T)$ appears nontrivial at this point.

## Data Availability

This article has no additional data.
